# Recent Advances of Pervaporation Separation in DMF/H_2_O Solutions: A Review

**DOI:** 10.3390/membranes11060455

**Published:** 2021-06-20

**Authors:** Zongqi Zhang, Siquan Xu, Yuanfeng Wu, Shengbin Shi, Guomin Xiao

**Affiliations:** School of Chemistry and Chemical Engineering, Southeast University, Nanjing 211189, China; 230198252@seu.edu.cn (Z.Z.); siquanxu@ahau.edu.cn (S.X.); 230189252@seu.edu.cn (Y.W.); 230198263@seu.edu.cn (S.S.)

**Keywords:** DMF, pervaporation, mixed matrix membranes, polymeric membranes, zeolite membranes

## Abstract

N,N-dimethylformamide (DMF) is a commonly-used solvent in industry and pharmaceutics for extracting acetylene and fabricating polyacrylonitrile fibers. It is also a starting material for a variety of intermediates such as esters, pyrimidines or chlordimeforms. However, after being used, DMF can be form 5–25% spent liquors (mass fraction) that are difficult to recycle with distillation. From the point of view of energy-efficiency and environment-friendliness, an emergent separation technology, pervaporation, is broadly applied in separation of azeotropic mixtures and organic–organic mixtures, dehydration of aqueous–organic mixtures and removal of trace volatile organic compounds from aqueous solutions. Since the advances in membrane technologies to separate N,N-dimethylformamide solutions have been rarely reviewed before, hence this review mainly discusses the research progress about various membranes in separating N,N-dimethylformamide aqueous solutions. The current state of available membranes in industry and academia, and their potential advantages, limitations and applications are also reviewed.

## 1. Introduction

N,N-dimethylformamide (DMF) as an excellent solvent is applied in various industries (e.g., pharmaceutical and petrochemical industries), formation of pesticides and manufacture of synthetic leathers, fibers, films and surface coatings [1]. At present, one-step DMF synthesis is widely adopted in China, in which dimethylamine, carbon monoxide and sodium methanol are separately applied as the raw materials and the catalyst during the reaction. DMF is applied: (1) as an analytical reagent, solvent and important intermediate in organic synthesis; (2) as an extractant and raw material for medicine and chlorphenamidine; (3) as a gas absorber in the petrochemical industry. After being used, DMF can form 5–25% spent liquors (mass fraction) [2] that are unrecyclable with distillation. The hazardous DMF wastewater can damage human health through breathing and skin contact, and cause eyes irritation, hematopoietic or liver dysfunction after long-term contact or inhalation. DMF in industrial wastewater is chemically stable and can be hardly biodegraded. Moreover, the recommended maximum admissible mass concentration of DMF in surface water is 25 mg/L in China. Hence, it is necessary to recycle DMF from waste from the aspects of environmental conservation and capital reduction.

**Figure 1:**
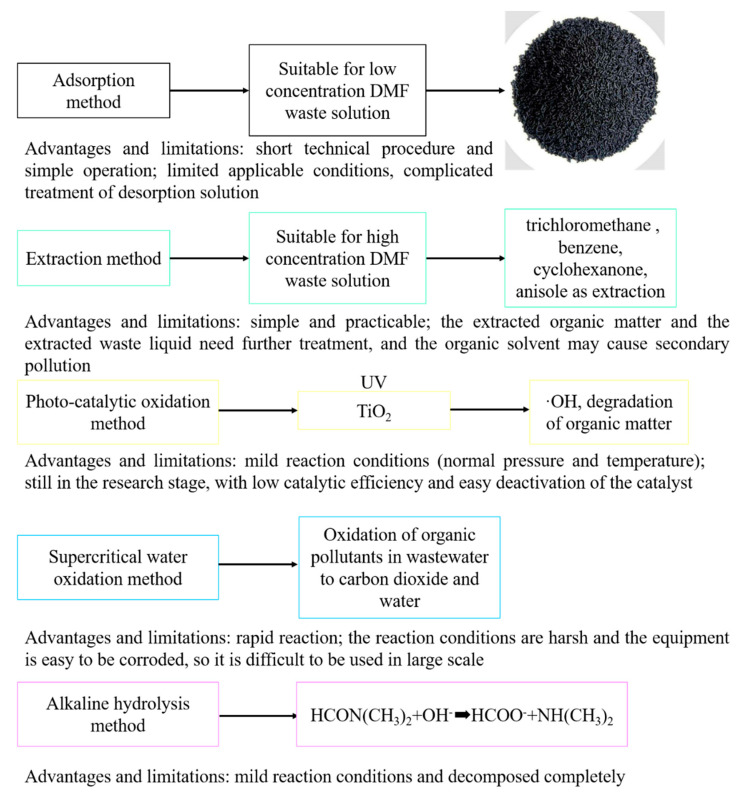
Some existing technologies used for DMF separation and their relative advantages and drawbacks.

At present, the wastewater containing DMF has been treated by various methods, including biological methods [3], physicochemical methods [4] (adsorption and extraction) and chemical methods (catalytic oxidation [5], supercritical water oxidation [6] and alkaline hydrolysis). The above methods are suitable for treatment of wastewater with different concentrations of DMF. Firstly, one of the biological methods is a conventional aerobic activated sludge process [7,8,9]. Biological process is the most widely used method in wastewater treatment because of its mature process and low operating cost. However, biological treatment of DMF wastewater is always limited by the long treatment time and incomplete degradation. Biological treatment will be negatively impacted by the microbial poisoning caused by DMF. Secondly, physicochemical methods are economical and rapid pretreatment methods for DMF removal and, especially, extraction and adsorption methods are commonly used in industry. Low-DMF wastewater can be adsorbed by activated carbon, while high-DMF solutions can be separated by trichloromethane, benzene, cyclohexanone, anisole and other extractants [4,10]. Thirdly, among all chemical methods suitable for industrial application alkaline hydrolysis is based on the principle that amides can be broken into corresponding amines and salts in a strong base solution [11]. Besides, this method requires mild reaction conditions with DMF completely decomposed, which deserves further research in industrial application (Figure 1).

Conventional separation methods [12], such as distillation, adsorption and supercritical water oxidationare faced with four limitations: (1) addition of the entrainer or extractant, which may be carcinogen [13] like benzene; (2) intensive consumption and high costs; (3) operational complexity and insecurity; (4) huge occupation areas of operating facilities. Hence, development of energy-efficient separation technology becomes a major concern for researchers.

**Figure 2:**
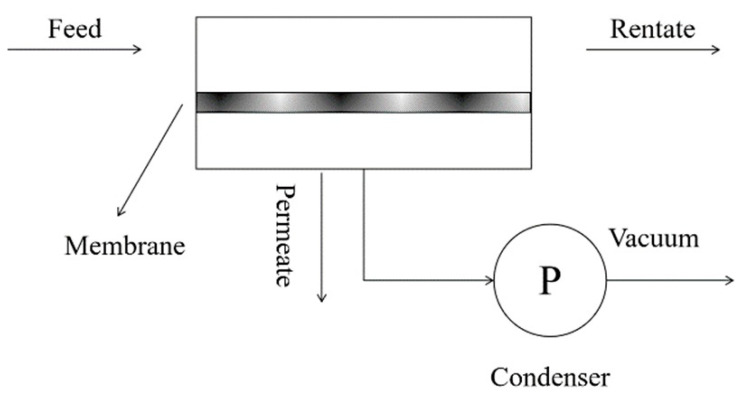
Graphical presentation of typical vacuum pervaporation process.

**Figure 3:**
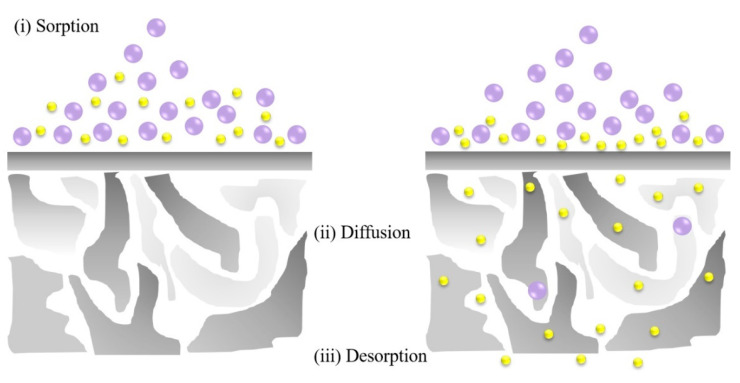
Graphical presentation of the solution–diffusion model.

Pervaporation (PV), an emerging technology, is a relevant part of energy conservation and environmental friendliness of membrane separation [14]. It is competitive over conventional separations in separating azeotropic mixtures, thermally sensitive compounds and organic–organic mixtures and in removing dilute organic compounds from wastewater [15,16,17,18]. Comparably, evaporation is the thermally based separation process, through which the solvent is separated from the non-volatile solute by phase transition [19]. At the molecular level, this latent heat reflects the energy required to break hydrogen bonds between water molecules, which is much greater than that required to move solute and water molecules, and to overcome their inherent tendency for a homogeneous state [20]. Since only a fraction of a mixture is vaporized, pervaporation requires lower temperature than conventional distillation, which is more energy-saving. This process can be divided into two parts: the upstream side of the membrane, which contacts with the feed liquid mixture, and the downstream side, which is applied with vacuum or sweep gas to generate a chemical potential difference, namely a diving force for the separation [21] (Figure 2). The separation mechanism of pervaporation predominantly relies on the preferential sorption and diffusion of the target component through the membrane [22,23,24] (Figure 3).

**Figure 4:**
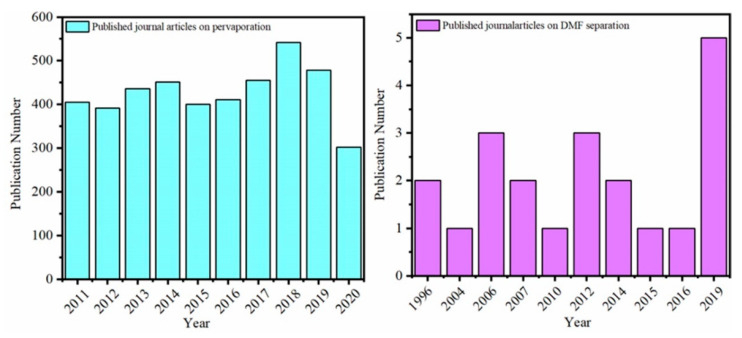
Recent published journal articles on pervaporation and DMF separation. Data were obtained from the Web of Science.

**Figure 5:**
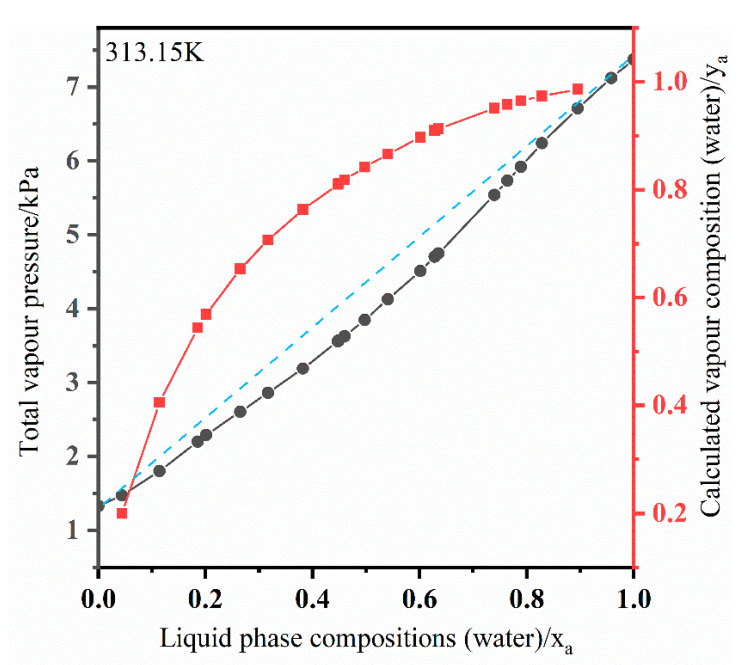
Vapor–liquid equilibria of DMF + water as a function of mole fraction of water.

Figure 4 illustrates the annual number of published articles on pervaporation or separation of DMF from water by pervaporation. In recent 10 years, about 400 published articles are about pervaporation, indicating this emerging technology becomes a research focus and concern of researchers. However, most membranes are applied to separate ethanol/H_2_O for commercial application, and only a few membranes are focused on DMF/H_2_O separation. In conclusion, the solubility of most polymers in DMF limits the selection and application of membrane materials. Up to date, research on DMF separation from water is limited and rarely reviewed. Figure 5 and Table 1 illustrate Vapor–liquid equilibria of DMF + water as a function of mole fraction of water [25].

Herein, a brief review presents the advancement in DMF/H_2_O pervaporation separation. This review discusses the research and progress of pervaporation on DMF/H_2_O, relevant limitations and challenges in polymeric membranes, mixed matrix membranes and inorganic membranes and fundamental theory and membrane fabrication procedures.

## 2. Theory of Pervaporation

Solution–diffusion is the worldwide accepted mechanism of mass transport through membranes, which was first proposed by Graham [26] based on his research on gas permeation through homogeneous membranes. As can be seen in Figure 3, the permeation is composed of following three fundamental processes: (1) sorption of solute molecules in the surface of the membrane; (2) diffusion of the molecules across the membrane; (3) desorption of the dissolved molecules in the other surface of the membrane. Generally, it is acknowledged that the thermodynamic equilibrium reaches immediately at the interface between feed side and membrane when feed mixture contacts with the membrane [26,27], therefore

(1) $$\frac{c_{i}}{c_{f}}=K$$

where *c_i_* and *c_f_* represent the concentration of component *i* in the membrane surface and the feed, respectively, and *K* is the partition coefficient, which is an intrinsic parameter dependent upon the interaction of the component *i* with the membrane. Molecule diffusion in micropore, which can be described by Fick’s first law [28],

(2) $$n_{i}=-D_{i}A\frac{d_{c_{i}}}{dx}$$

where *n* represents the permeation flux through the membrane; *D* is the diffusion coefficient; *A* is the efficient sorption area and *x* is the position variable along the transmembrane direction. By inserting Equation (1) into Equation (2) yields:

(3) $$n_{i}=-D_{i}KA\frac{d_{c}}{d_{x}}$$

where *c* represents the hypothetical liquid-phase concentration at position *x* inside the membrane. Meanwhile, the concentration of the component *i* can be expressed in its concentrations in the feed and the permeate side, respectively. Then, Equation (3) becomes [28]

(4) $$n_{i}=D_{i}KA\frac{\Delta c}{x}$$

where the diffusion, partition coefficient and membrane area are constant.

The permeation flux, the separation factor and pervaporation separation index (PSI) [29] can be obtained experimentally,

(5) $$J=\frac{Q}{A\cdot \Delta t}$$

(6) $$\alpha =\frac{y_{p}/x_{p}}{y_{f}/x_{f}}$$

(7) $$PSI=J_{t}\cdot \alpha$$

or

(8) $$PSI=J_{t}\cdot \left(\alpha -1\right)$$

where *J* is the permeation flux; Q is the quantity of the permeate collected in a time interval Δt; A is the effective membrane area; *α* is the separation factor; *x* and *y* represent the different components of the mixture, the subscript *p* and *f* representing permeate side and feed side, respectively. *J_t_* is the total permeation flux.

On the other hand, permeability (P) or permeance (P/*l*) reflects the driving force normalized productivity of a dense or an asymmetric membrane, respectively [23,30],

(9) $$J_{i}=\frac{P_{i}}{l}\left(f_{i,f}-f_{i,p}\right)$$

where *P_i_* is the permeability of component *i* across the membrane, *l* is the thickness of the selective layer, *f*_*i*,*f*_ and *f*_*i*,*p*_ are fugacities or partial vapor pressure of component *i* on feed and permeate sides of the membrane, respectively. The fugacity of component *i* in the feed side based on its liquid concentration can be determined by:

(10) $$f_{i,f}=x_{i}y_{i}P_{i}^{sat}$$

where *x_i_*, *y_i_* and $P_{i}^{sat}$ are the mole fraction, activity coefficient and saturated vapor pressure of component i in the feed, respectively. By inserting Equation (9) into Equation (10) yields:

(11) $$J_{i}=\frac{P_{i}}{l}\left(x_{i}y_{i}P_{i}^{sat}-f_{i,p}\right)$$

Considering pervaporation and flash distillation options, a simple method to evaluate pervaporation and elementary flash distillation is as follows [31]:

(12) $$\mathrm{MFLI}=\frac{y_{i}^{PV}}{y_{i}^{D}\left[VLE\right]}$$

where MFLI means membranes flash index, $y_{i}^{PV}$ is the permeate concentration and $y_{i}^{D}\left[VLE\right]$ is the equilibrium distillation value.

In theory, three types of effects, coupling effect, swelling effect and concentration polarization effect often exist in pervaporation. Coupling effect refers to that the diffusion rate of one permeant is dependent on the others. The formation of associated molecules, restricted in the membrane partially, result from attractive molecular interactions between liquid components, thus causing a mutual drag among them [32,33,34]. Coupling effect can be alleviated by improving the preferential affinity of membranes for the particularly component. Swelling effect lies in the fact that the exposure of membranes in solution over time leading to a decrease in the mechanical strength and the permselectivity [35]. Swelling of membranes can be mitigated by increasing the content of rigid fillers in the polymer matrix. Concentration effect derives from the fact that when the different components are carried to the surface of the membrane, the preferential adsorbed component permeate the membrane first while the other components in the feed side accumulate, which cannot be balanced by back diffusion of rejected component, hence the concentration of rejected components further forming excessive cumulation [36]. Concentration polarization effect, normally as a priority to be considered, would provide additional impediment to the desired components to permeate. One effective method to restrain concentration polarization effect is to increase the flow rate of feed, which can accelerate molecular motion to facilitate the diffusion of rejected component to alleviate the enrichment of that concentration in the surface of the membrane.

## 3. Common Fabrication Methods of Membranes

### 3.1. Solution Casting

One of the most common methods to synthesize flat-sheet membranes is solution casting, which applies in various situations. The polymer and potential additives such as inorganic fillers are initially dissolved in a solvent to form a solution and then the mixtures are casted onto a flat surface such as petri dish or stainless steel plate. The solvent can be removed through evaporation and/or phase inversion processes. Through the casting method with or without the participation of porous support, the multilayer film can be successfully prepared. By removing the solvent slowly and completely through evaporation, a high-density cell membrane can be prepared. In contrast, when the solvent removal process involves a phase inversion process, an asymmetric membrane with interconnected cell structures can be obtained by immersing in a non-solvent bath. In order to achieve the purpose of preparing the top dense layer, an effective way is to add a highly volatile solvent to the casting solution and perform phase inversion after evaporation. For the instance of mixed matrix membranes (MMMs), to prevent the agglomeration phenomenon of fillers, the mixtures are commonly treated by thorough stirring and sonication before casting. Subsequently, MMMs are synthesized by the similar procedures as aforementioned. For example, poly (vinyl alcohol) with acrylamide to form a polymeric membrane is prepared in this way.

### 3.2. Hollow Fiber Spinning

Compared with flat-sheet membranes, hollow fiber membranes with the shell feed mode exist the following advantages such as higher packing density, self-supporting structure and self-containing vacuum channel. For the hollow fiber spinning process, it contains a large number of process parameters, which run through the entire spinning process such as coating formulation, coagulation chemistry, spinneret design and spinning conditions (air gap, temperature and take-up speed). During spinning process, the virgin fiber combines with the coagulant to form the membrane through phase inversion. Coagulation process occurs immediately at its internal surface after the virgin fiber coming out from the spinneret due to the simultaneous extrusion of the polymer dope and the bore fluid in the lumen side of the nascent fiber. Meanwhile, since the existent of humidity in air, when the nascent fiber passes across the air gap zoom, partial coagulation occurs at the outer side of the fiber. The whole phase inversion process is completed once the fiber is fully precipitated in the external coagulation bath. Varying compositions of the spinning dope, bore fluid and external coagulant and take up speed, these factors can determine the thickness and morphology of the selective layer. Though the complexity of hollow fiber spinning increases as the spinning method advances from single-layer to dual-layer coextrusion. The dual-layer hollow fibers exhibit the benefits of capital reduction and independence in customization of materials and morphology for the selective and supporting layers [37,38].

### 3.3. Solution Coating

Solution coating is a common method [39,40,41] to synthesize composite membranes for the deposition of a thin selective layer on top of microporous substrates or supports, which can be in the form of either flat-sheet, hollow fiber or tubular module to overcome fouling or improving solvents resistance and to enhance membrane efficiency [42,43,44,45,46]. For the sake of minimizing the substructure resistance, fully porous substrate will be preferred so that the coated selective layer mainly determines the membrane resistance [47,48,49,50]. It should be noted that to hinder from invasion of the coating solution, the necessity for the pore size distribution of the substrate surface is preferentially sharp and free of large defects. Occupying the pore structure with other low boiling point solvent, which is easy to remove and immiscible with the coating solvent prior to the coating process, which is called the prewetting process, can minimize the intrusion [47,49,50]. Then the prewetting solvent is removed by drying followed by the coating procedure to obtain the coated membrane. The most commonly applied configuration is the flat-sheet membrane through solution coating, due to the fact that the fiber with small diameters cannot be coated uniformly, which imposes a negative effective on the pervaporation separation process. For example, chitosan/PTFE membrane prepared from casting a γ-(glycidyloxypropyl)trimethoxysilane (GPTMS)-containing chitosan solution on poly (styrene sulfuric acid) grafted expended poly(tetrafluoroethylene) film surface.

### 3.4. Physicochemical Modifications

In order to increase the performance and stability of PV membranes, post-modification processes are generally adopted. In terms of the different separation system, PV membranes should exhibit the affinity to the particular component such as hydrophilic or organophilic. Incorporating or grafting appropriate functional groups into the polymer chains can be utilized to modify the membrane hydrophilicity and hydrophobicity, in order to improve the affinity between water or organic molecules and the membranes [51]. In addition, potential defects on the selective layer of the membranes can be alleviated by postannealing process [51]. For example, in PTFE-g-PSSA membrane synthesis, HCl(aq) solution was adopted to convert the sodium sulfonate groups to sulfonic acid groups to obtain high hydrophilicity and higher permeation flux.

## 4. Polymeric Membranes

Polymeric membranes are obtained by organic polymer crosslinking to form a series of chains and possess channels, which the molecule can transmit [52]. They characterize high-selective sorption and diffusion properties depending on their inter- and intramolecular structure. Generally, they are composed of rigid chain polymers capable of ionic dipole interaction or hydrogen bonding with water. The hydrophilic polymer membrane acts as a molecular sieve, which tends to be durable to water while absorbing water molecules preferentially over other molecules in the process stream. Semenova et al. [53] pointed out that a high sorption center is necessity for the polymer with high selective water permeation, which should have mutual effects with water by dipole–dipole actions, ion–dipole actions (in the case of a polyelectrolyte) and/or hydrogen bonding. In consequence, it is often desirable to select a membrane whose polymer chain possesses one of these characteristics or modifying membranes existed involving such features.

At present, the research starts with homogeneous polymers has been developed to improve the separation performance by modifying the properties of polymer in various ways. The characteristics of the polymer selected, such as separation performance, can be changed by regulation of the cross-linking degree or alteration of the affinity for water. Subsequently, a large quantity of different experiments for a certain system would be carried out for the purpose of screening out the optimum membrane with appropriate flux and separation factor. The advantage of an asymmetric membrane is that it can provide a thin layer of skin and a porous bottom surface. Therefore, while providing a dense and uniform membrane with a high flux ratio, a thin layer of composite membranes active on the support of different polymers also enables high flux to be achieved while maintaining high selectivity to the required permeation [52].

Aminabhavi and Naik [54] applied grafted copolymeric of poly (vinyl alcohol) with acrylamide (PVA-g-AAm) to water-dimethylformamide (DMF) mixtures. They prepared three membranes: (1) pristine PVA; (2) PVA with 48% grafting; (3) PVA with 93% grafting. The results indicated that selectivity was positively correlated to the degree of the membrane grafting through calculating the membrane-solvent interaction parameter, χ_ip_, using the following equation:

(13) $$\;\chi_{\mathrm{ip}}=0.34+\frac{V_{i}{\left(\delta_{s}-\delta_{p}\right)}^{2}}{\mathrm{RT}}$$

where *δ_s_* and *δ_p_* are the solvent and polymer solubility parameters, respectively. R is the gas constant and T is the absolute temperature. However, flux showed no considerable variation with the extent of grafting. The results of swelling degree were also positively correlated to the degree of the membrane grafting. After calculating Arrhenius activation parameters, the temperature dependency of the permeation flux, diffusion coefficient, and separation selectivity followed the Arrhenius trend.

Kurkuri and Aminabhavi [55] applied polyacrylonitrile (PAN) to grafting poly(vinyl alcohol) (PAN-g-PVA). They adopted the same analysis method, calculating χ_ip_ and diffusion coefficient D, and came to the similarly conclusion. It is noteworthy that polymer is hydrophilic after grafting, thus it preferentially interacts with water molecules more than DMF [55] by comparing the size of the values of diffusion coefficient D.

Devi [13] prepared poly(vinyl alcohol) (PVA)/poly(acrylic acid) (PAAc) blend membranes followed by crosslinking with glutaraldehyde (GA). With comparison to pristine PVA/PAAc, modified PVA/PAAc exhibited a higher selectivity and preferable thermal stability and optimum ratio was 9:1 (PVA:PAAc). It concluded that flux increased with enhancement of water concentration of the feed mixture on account of internal plasticization effect, whilst the flux of DMF was lower attributing to its larger molecular size than water. In addition, compared with water, DMF exhibits comparatively lower chemical affinity to PVA/PAAc membrane, hence better selective separation of water can be obtained, which is superior in the present membranes. With calculation of determination of crosslink density (υ_c_) and molar mass between crosslinks (M_c_) by dynamic mechanical and thermal analyzer (DMTA), the results implied that crosslink density decreases with an enhancement in PAAc content of the blend with the fact that lower the crosslink density, higher will be the elastically effective chain length (M_c_) and more flexible will be the film. Moreover, the operation temperature was 30 °C, which reduced the operation cost.

According to Ray [56], crosslinked copolymer membranes were synthesized by solution polymerization with arcylamid (AM) and 2-hydroxyethyl methacrylate (HEMA) to separate DMF/H_2_O solutions. In consideration of the membrane polymer dissolving in polar organic or being disintegrated, Ray [56] enhanced the stability of the membrane. The results showed that the degree of crosslinking and water selectivity of the membranes were positively correlated to the HEMA content in the membrane. However, the experiments were conducted under the condition of the concentration range of 0–13.07 wt % water in feed side, which means the performance of the membranes not being investigated in the terms of high concentrations of water (i.e., 90 wt % water in feed).

Das and Banthia [57] reported that they used polyurethane urea (PUU) and poly (methyl methacrylate) (PMMA) to synthesize a interpenetrating network (IPN) membrane, which is preferentially selective to DMF. They first synthesized polyurethane prepolymer, then synthesized a uniform mixture with azobisisobutyronitrile (AIBN) as an initiator. They synthesized three membranes with different content of PMMA, which were 8.51%, 16.99% and 26.62%, respectively and the third membrane (PUU-PMMA-3) performed the best performance among them. The swelling of the membrane could be up to approximately 60% in 100% DMF solution, which reflected the highly selectivity towards DMF than water. Simultaneously, polymer-solvent interaction parameters, χ_ip_, which the values of polymer and water were practically twice than those of polymer and DMF, also interpreted the same conclusion. The close solubility parameter values of DMF and membrane supported the affinity of the membranes towards DMF, which indicated the possibility of IPN membranes application in DMF recovery. This research is the only reported membrane currently that had priority for DMF permeation, which will develop a pathway for prospective research.

**Figure 6:**
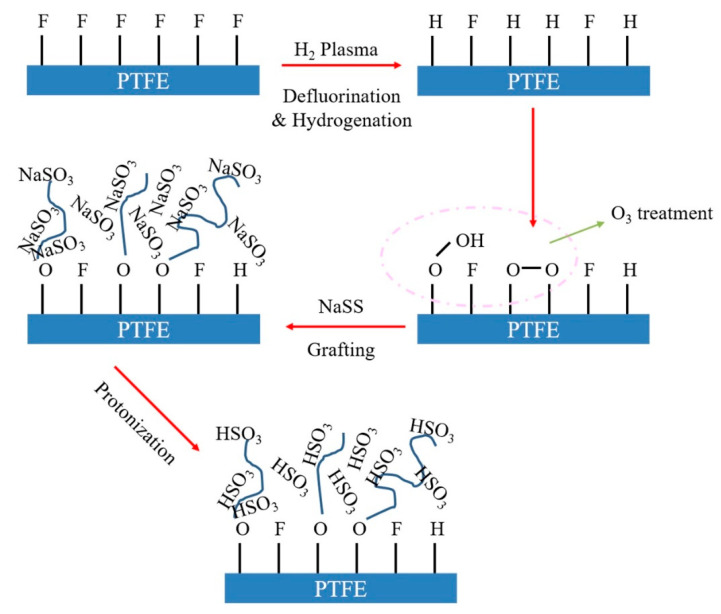
Fabrication procedures of the PTFE-g-PSSA membranes.

Tu et al. [58] prepared poly(tetrafluoroethylene) (PTFE) membranes performed by means of combined hydrogen plasma and ozone treatment and surface-initiating grafting polymerization in which sodium 4-styrenesulfonate (NaSS) was used as monomers. After that the PTFE-g-NaSS membrane was further treated with 1 M HCl(aq) solution at room temperature to convert the sodium sulfonate groups to sulfonic acid groups (PTFE-g-PSSA). With treated by hydrogen plasma, some C-F bonds with high bonding energy difficult to be treated by ozone on PTFE membrane surface were devoted to C-H bonds. Then, the C-H bonds served as reactive cites under ozone treatment to form peroxide groups. After that, NaSS on PTFE membranes surface triggered surface-initiating polymerizations generating hydrophilic chains consequently leading to the incensement of the surface hydrophilicity. Finally, with protonation, the sulfonated groups of PTFE-g-PNaSS were converted to sulfonic acid groups (PTFE-g-PSSA) (Figure 6). The polymerization rendered the membranes high hydrophilicity and higher permeation flux with a water contact angle of 38° and permeation flux of 345 g·m^−2^·h^−1^ while those without protonation with an angle of 68° and flux of 308 g·m^−2^·h^−1^. It is notable that the swelling degree of the membrane in DMF solutions (90 wt % of DMF content) was 0.34% less than 0.4% indicating its resistance to swelling and application potential for separating DMF. It is also noteworthy that the separation factor of this work was up to infinite, namely, 100 wt % of water in the permeating side, which is deliberate.

Liu [59] and coworkers synthesized chitosan/PTFE composite membranes prepared from casting a γ-(glycidyloxypropyl) trimethoxysilane (GPTMS)-containing chitosan solution on poly(styrene sulfuric acid) grafted expended poly(tetrafluoroethylene) film surface. They utilized GPTMS in chitosan solution to form chitosan-silica nanocomposites, then casted chitosan/GPTMS solutions onto PTFE-g-PSSA film surface to obtain the chitosan-5/PTFE composite membrane, where 5 means the mass fraction of GPTMS was 5%. The optimum layer thickness of the chitosan layer as about 1.26 μm, which was tailored with casting solution concentration and the optimum concentration was 3 wt % chitosan casting solution. Membrane life experiments lasted 45 days suggesting that the fabrication method can definitely facilitate long-term operation stability of membranes in the pervaporation dehydration process.

Solak et al. [60,61,62] prepared a series of blend membranes from sodium alginate (NaAlg). First, they synthesized NaAlg membranes crosslinked with calcium chloride. Considering the water solubility and excess hydrophilicity of NaAlg, it is suitable to modify the membrane material with different form of polymer blends. Then, they used N-vinyl-2-pyrrolidone (NVP) grafting NaAlg under atmosphere of N_2_ with benzophenone. The graft polymerization was carried out by irradiating with UV light. It could conclude that the permeation rate of grafted NVP on alginate membranes was higher than that of NaAlg membranes and sorption selectivity was the main factor affecting the separation of DMF/water mixture. It could also draw to that with the increase of operating temperature, the permeation flux increased and the separation coefficient decreased. However, in all methods, with the increase of DMF feed content, permeation flux decreased and separation factor increased. They detected that increase in the operating temperature increased the permeation flux whereas the separation factor as well. Third, they prepared NaAlg/polyvinyl pyrrolidone (PVP) blend membranes and investigated the performance in separation of aqueous DMF solutions in the concentration range of 0–100 wt %. NaAlg/PVP blend membranes were prepared in different ratios and crosslinked with calcium chloride. The conclusion could be drawn that blending of PVP with the NaAlg membranes had higher permeation flux than that of pristine NaAlg membranes and higher separation factors than that of NaAlg-g-NVP membranes. Due to the cheap and easy preparation method, as a result it could be said that NaAlg/PVP blend membranes had good separation performance, which revealed higher selectivity and was more suitable than the NaAlg-g-NVP.

John and Kamalesh [63] adopted per-fluoro-2,2-dimethyl-1,1,3-dioxole copolymerized with tetrafluoroethylene (PDD-TFE) to fabricate perfluoropolymer membrane in dehydration of aprotic solvents. At 50 °C, the maximum water flux values of 77 g·m^−2^·h^−1^ was obtained in separating DMF–water mixtures. Compared with NaA zeolite membranes, the perfluoro membranes presented better performance (separation factors were found to be on the order of thousands to tens of thousands) in separating aqueous solutions of aprotic solvents. Furthermore, such cost efficiency thin composite membranes can then be commercialized in successful PV systems.

Ma et al. [64] reported pyromellitic dianhydride (PMDA) and 3,3′,4,4′-benzophenoneteracarboxylic dianhydride (BTDA) based polyimide membranes and investigated the effect of chain structure on the solvent resistance in aprotic solvents. They chose various aromatic p, p’-diamines including 4,4′-diaminodiphenyl ether (ODA), diaminodiphenylmethane (MDA), 2,2-bis [4-(4-amlnophenoxy) phenyl] propane (BAPP) and benzidine (BZD) as the diamine monomers, forming symmetric and straight chains with strong interactions between them. They found that the chain structure and the elasticity modulus of the PI membrane influenced the swelling degrees in DMF. As for the PMDA-based PI membranes, the addition of -O-CH_2_ and -CH_2_-flexible bonds decreased the rigidity of the PI main chain and inhibited close packing of the chains, which reduces the interactions between chains and leads to reduce the solvent resistance [65]. According to the conclusion, higher interchain distances of PMDA based membrane resulted in a weak molecular chain interaction and straightly stiff backbone and close interchain distance led to the lowest swelling of BDTA based membrane. When the molecular chain interaction was the weakest, it led to the loose accumulation of the chain, resulting in the lowest tensile strength. They postulated that the solvent resistance of the PI membranes with various chain structures served as a function of the elasticity modulus, which swelling degrees decreased linearly with an increase of the elasticity modulus. The water contact angles of all membranes were approximately 70°, manifesting that the surface of the membranes was relatively hydrophilic. However, the stability and performance over extended time of hybrid membranes were only implemented in N, N-dimethylacetamide (DMAc) at 500 Pa and 50 °C with high flux and a small change in the separation factor demonstrating that the excellent separation stability in DMAc solution. However, the stability in DMF of PI hybrid membranes was not available.

The pervaporation performance of polymeric membranes reviewed in this section can be listed in Table 2.

Due to possessing the following merits, low capital, easy access to raw material, simple processability, good mechanical stability and adjustable conveying performance, polymers are initially and most widely used as pervaporation membrane materials. However, the development of polymeric membranes is restricted by their limitations such as poor antipollution properties, low chemical and thermal stability and in particularly the intrinsic trade-off effect between permeability and selectivity. The future development in polymeric membranes would be focused on the raw material selection, polymer synthesis and modification process.

The modification of polymer properties through polymer blends has been increasingly employed to obtain inexpensive materials with enhanced characteristics. As a result, the blend properties of membranes own the intrinsic chemical, physical and mechanical properties of each polymer [66]. However, there are also some intrinsic limitations, which adversely affected the usage of pristine polymers. At present, polymer blending with PIM-1 of superior permeability that can overcome the limitations have received attention, which is one of the straight forward strategies to improve the overall performance. Nevertheless, the major limitation in polymer blends is the phase separation behavior, which deserves further investigation. It should be highlighted that it is relatively challenging to develop an ideal, fully miscible blend membrane since the two polymers have different solubility parameters and intrinsic characteristics. The in-depth understanding of the structure–property relationship between homogeneous and heterogeneous blend membrane is important and it should be predicted through the development of transport models such as using the molecular dynamic (MD) simulation. Through that, one can easily manipulate the properties of the blends based on the proper selection of polymers and their compositions. Moreover, the polymer blend can be used as a parent material to combine with other modifications such as cross-linking, hybrid, composite, doping, incorporated with compatibilizer or nanofillers to form mixed matrix membranes [66].

In a nutshell, the polymer blend is well recognized as the most viable, cost- and time-efficient strategy to the market. Moreover, based on the remarkable extent of research, polymer blend has been proven as a promising strategy. With more pioneering work towards in-depth understanding and rational design of materials, membrane separation with desirable performance could be realized.

## 5. Mixed Matrix Membranes

Mixed matrix membranes (MMMs) refer to inorganic fillers dispersed in a polymeric matrix, which is a type of hybrid organic–inorganic membranes [14,67,68]. MMMs, which combine the strengths of inorganic and polymeric membranes, were first patented by Kulprathipanja et al. in 1988 [69,70]. To improve the trade-off resistance, which means a balance between permeability and selectivity [71], researchers used various fillers in MMMs for pervaporation, including zeolites [72], silica [73], carbon nanotubes (CNTs) [74], multiwalled CNT (MWCNTs) [75,76,77], metal organic frameworks (MOFs) [78,79], zeolite imidazolate frameworks (ZIFs) [80,81] (a subclass of MOFs), covalent organic frameworks [82], graphenes [83], graphene oxides (GOs) [84], porous organic cages (POCs) [85], polyhedral oligomeric silsesquioxanes (POSSs) [86] and metal oxide nanoparticles [87]. The addition of an inorganic material can strengthen the mechanical properties of the membrane and reduce the free volume of molecule diffusion. However, only zeolite membranes have been reported currently on DMF/H_2_O separation, other fillers may be prospective for DMF wastewater reclamation in future research.

**Figure 7:**
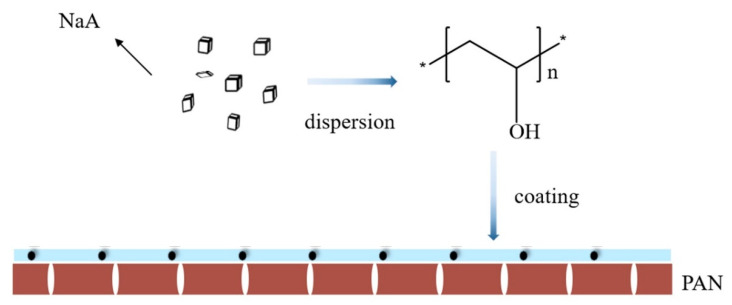
Fabrication procedures of the NaA/PAN membranes.

Shao and her coworkers [12] prepared NaA zeolite membranes by dispersing NaA zeolites homogenously in PVA solutions. Then, the membrane solution was coated on the polyacrylonitrile (PAN) substrate (Figure 7). They focused on the effect of the times of membrane casting on pervaporation performance and concluded that the optimum times of casting was 3. The mass fraction of NaA in casting solution was 10% and operation temperature was 24 °C, which was at room temperature meaning without extra heating supplement. The results indicated that the permeation increased with the enhanced temperature, which ranges from 18 to 42 °C. The permeation flux of NaA/PAN membrane reached 1.84 kg·m^−2^·h^−1^ and the separation factor was 11.5 when the feed mass fraction of DMF was 20%, the feed flow rate was 1.5 m^3^/h and the pressure at the permeate side was 500 Pa.

Ma [88] and Matsuyama synthesized UiO (University of Oslo)-66/polyimide (PI) membrane with the mass fraction of UiO-66 was 2%, which is a new breakthrough in the realm. They added UiO-66 prepared by hydrothermal synthesis method into the polyamic acid (PAA) solution. After stirring several hours, the homogeneous solutions were spin-coated onto the α-Al_2_O_3_. Then, the UiO-66/PI hybrid membranes were obtained by the thermal imidization process. They focused on the loading of UiO-66 (0, 1, 2, 3 and 4 wt %) and concluded that 4 wt % is the optimum loading. With the water and DMF adsorption isotherms of UiO-66, the water uptake reached to 200 mg/g at P/P0 = 0.5, while the DMF uptake was only 20% indicating that the pore size of MOF benefiting for water molecule transporting. The water contact angle of UiO-66(2 wt %)/PI membrane was 56°, which was distinctly smaller than that of pristine PI membrane, which was 73°. All hybrid membrane possessed more hydrophilic than that of the pristine PI membrane, which indicates a better affinity with water. The UiO-66 addition enhanced the PI chain packing, hydrophilicity and solvent resistance, in comparison with the pure polyimide membrane. The UiO-66/PI hybrid membranes with 2 wt % incorporation exhibited high hydrophilicity and excellent swelling resistance as a result of complexation of UiO-66 and PI chain leading to the excellent dispersion. The experiment was carried out at the temperature spanning from 303 to 343 K, whilst the flux increased an order of magnitude from 65.3 to 489.8 g·m^−2^·h^−1^. It could conclude that evaluated temperature facilitated water and solvent molecule permeation and thermal motion of polymeric chains and increase in fractional free volume in the membrane bulk. It is notable that the stability over time was not mentioned in the article.

The pervaporation performance of MMMs reviewed in this section can be listed in Table 3.

Though MMMs inherit distinct superiority over polymeric membranes and inorganic membranes, three critical issues should be noticed, including distribution of fillers, hierarchical structures [89] and interaction between polymer and fillers phases. Interfacial voids, rigidified polymer chain layer and pore blockage [69,90] may inevitably result from the differences in physicochemical properties between polymer and fillers and clustering. An ideal synthesis method to improve separation performance should encompass three characteristics. First, inorganic fillers can be dispersed homogeneously in the polymer matrix. Then, the fabrication should be characteristic of flexibility and precision in control. Finally, a comprehensive range of fillers with multiscale structures and multiple functionalities should be easily available. In other words, the ideal fabrication method must be facile, controllable, efficient and versatile [67].

MOFs have been widely investigated in recent years as some of the most promising materials due to their stable frameworks. Some aluminum MOFs, including MIL-53 [91,92], MIL-96 [93], NH2-MIL-101 [94,95], CAU-21-ODB [96] and NH2-MIL-53 [94,97], are incorporated into polymer matrices. The successfully application of various aluminum MOFs such as DUT-5 [98], CAU-1 [99] and MIL-53 [100] in MMMs contributes into flux increment in the pervaporation study. However, these MOF-based membranes are only applied in ethyl acetate–water [101], dehydration of acetic acid and ethanol [102]. These facts indicate that MOF-based membranes will be promising materials and are broadly prospective in future research.

## 6. Inorganic Membranes

Inorganic membranes can be divided by the surface structure into zeolite membranes and density membranes. For the application of zeolite in separation and catalysis, zeolite membranes have attracted much attention from current research and development. Compared with polymeric membranes, zeolite membranes demonstrate four advantages [103,104,105,106]: (1) higher swelling resistance against solvents; (2) uniform, molecular-sized pores that cause significant differences in transport rates for some molecules, and allow molecular sieving in some cases; (3) higher chemical stability in most cases, especially in low pH mixtures or polar solvents; (4) higher thermal-stability (up to 1270 K [107]). Nevertheless, the obvious inherent restrictions, high costs, brittleness and difficulty in fabrication of large defect-free membranes faced by zeolite membranes should be urgently overcome [108].

Zeolite membranes can be divided into following types: LTA-type zeolite [109,110], ERI-type zeolite [111], FAU-type zeolites X and Y [112,113], MOR-type [114], CHA-type zeolite [115,116], FER-type zeolite [117], MEL-type [118], MFI-type ZSM-5 and silicalite-1 [119,120] and amorphous zeolite [121,122,123]. Zeolite is a kind of crystalline microporous material containing molecular size channels and pores. It is composed of tetrahedral building units of TiO_4_ (T = Si and Al) and is connected by oxygen atoms to form a one, two or three-dimensional network [124]. The hydrophilicity/hydrophobicity, the ion-exchange capacity, the catalytic and the acid stability are affected by the Si/Al ratio. There have also been some methods to control membrane properties including silylation to decrease pore size [125] and to increase hydrophobicity [126,127] and chemical vapor deposition (CVD) [128], atomic layer deposition (ALD) [129] or coking [130] to fill non-zeolite pores. The commonly used for supports are alumina supports typically having pore diameters between 5 (γ-Al_2_O_3_) and 200 nm (α-Al_2_O_3_) and stainless steel supports having pore diameters between 0.5 and 4 μm [103].

Shah et al. [131] used commercial zeolite NaA membranes to separate dimethylformamide–water mixtures and discussed the different transport mechanisms how to impact the pervaporation flux and selectivity particularly. On the premise of solution–diffusion mechanism, the mass transport can be elucidated by the Maxwell–Stefan equation [132]. Through some simplifying assumptions, such as no counter-diffusion of molecules, the Maxwell–Stefan equation can be transformed to a linear relationship [24]. However, the ionic interactions influence simultaneously the sorption and diffusion of water in the membrane. It can be envisioned that with regard to the zeolite membranes, the solution-diffusion mechanism is divided into two steps: the first step is that water molecules preferentially sorb at the cage mouth and the second step is that the water molecules adsorbed diffuse across the active layer. However, it may also need to be considered that partial molecular sieving effects and permeation through non-zeolitic pores with regard to solvent molecules. It was noteworthy that the water flux for the DMF–water system decreased rapidly with an increase in the feed DMF concentration. Considering that DMF is an aprotic solvent with a high dipole moment, they contributed it to the strong sorption of DMF on the ionic sites in the zeolite membrane resulting in a blocking of the water sites and therefore the lower water flux.

Sommer and Melin [124] investigated the separation performance of commercial A- and T-type zeolites membranes and microporous silica membranes from ECN and Pervatech. According to the research, it was observed that a comparatively high selectivity of DMF was beneficial to water permeation, and according to the measurement of standard reference test conducted after each experiment, DMF caused a considerable decrease in the permeability of all membranes. They concluded that the membrane was fouled irreversibly owing to the immersion of membrane surface in the solvent molecules. This effect can be currently explained by two different viewpoints. One is that highly polar molecule (indicated by a high dipole moment or dielectric constant) interacts with the icon sites in the zeolite cage strongly, which leads to competitively adsorption of solvent molecules [131,133]. The other is that high boiling point solvents, which permeate through the membrane result in the blockage of the porous support. The above two points both lead to the decline of permeation of flux. After pure water pervaporation experiments at the temperature spanning from 90 to 110 °C, the silica membrane was damaged leading to a decrease in selectivity, which silica is instable at higher temperatures. While A- and T-type zeolite membranes showed no sign of thermal degradation up to 150 °C and could withstand operation pressure up to 30 bar. They characterize higher separation factors combined with lower permeation flux than the two silica membranes. Compared to polymeric membranes, the inorganic membranes exhibited excellent chemical stability in aprotic solvents like DMF and in acids and bases whose pH values range from 3 to 11. Meanwhile, thermal stability of inorganic membranes several times higher than that of polymeric membranes.

**Figure 8:**
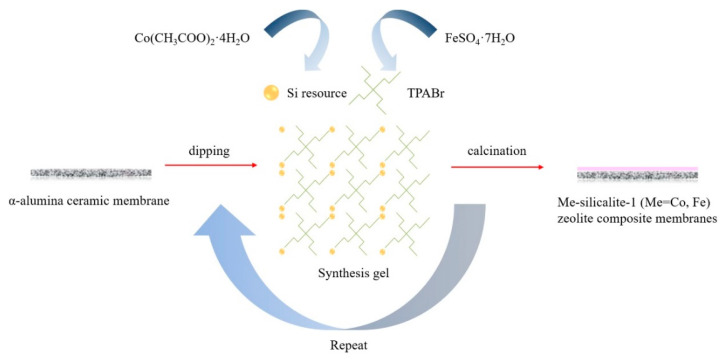
Fabrication procedures of the Me-silicalite-1 (Me = Co and Fe) zeolite composite membranes.

Shao [2] and her colleagues reported a Me-silicalite-1 (Me = Co and Fe) zeolite composite membranes supported on α-alumina discs prepared by seeded secondary growth method (Figure 8). They aimed to substitute metal ions (Co and Fe) into the zeolite framework to improve the hydrophobicity of the membranes. Comparing the pervaporation activation energy, the energy of silicalite-1 modified by Co and Fe were less than that of pristine solicalite-1. The results indicated that metal doping could change the membrane surface to organic permselectivity, which is conducive to the recovery of DMF solvent from dilute DMF waste water in industry. The permeation flux of the Co-silicalite-1 zeolite membrane and Fe-silicalite-1 zeolite membrane reached 0.66 kg·m^−2^·h^−1^ and 0.84 kg·m^−2^·h^−1^, the separation factor 4.4 and 2.9 when the feed mass fraction of DMF was 5% and feed temperature was 40 °C, respectively.

Ji and his coworkers [134] prepared a chabazite (CHA) zeolite membrane supported on external surface of yttria-stabilized zirconia (YSZ) hollow fiber. In consideration of the effective pore size of CHA membrane, which is 0.38 nm, it was feasible to apply it to organic solvent dehydration [135,136]. To enhance the permeation of pristine CHA membranes, they adopted yttria-stabilized zirconia (YSZ) with high loading density, thin tube thickness and low mass transfer resistance as support. It was noteworthy that the water flux permeation initially decreased and then stabilized after 24 h stabilization period, which contributed to the adsorption of DMF on membrane surface and in pores, and the water content in permeate stabilized at above 99%. The permeation flux of CHA zeolite membrane reached 5.7 kg·m^−2^·h^−1^ and the separation factor was 1180 when the feed mass fraction of water was 10%, the feed temperature was 75 °C.

Hasegawa et al. formed CHA zeolite membrane on the porous α-Al_2_O_3_ support with various Si/Al ratios [135,137,138,139,140]. Compared with Ji’s work, they obtained better separation selectivity factor, however, lower flux when CHA-type zeolite layer with Si/Al = 17.

The pervaporation performance of inorganic membranes reviewed in this section can be listed in Table 4.

For the zeolite membranes, it has been observed that the secondary growth method favors high reproducibility and good control over the membrane structure [142,143]. Recently, MOFs membranes with improved molecular separation property and permeation flux have been applied in compact devices. However, only a few reports were available about ZIF materials. This particular area can be exploited further in the future [144].

## 7. Perspectives

This review discusses the state-of-the-art in polymeric membranes, mixed matrix membranes and inorganic membranes in DMF–water separation. The market vacancy generated chemical and petrochemical industries bring a huge market for pervaporation separation, and organic solution separation will undoubtedly be the key for future research. More research in the following directions will be conducive to addressing this challenge. While a more diffusion-selective copolymer is synthesized, the synergy between the diffusion and adsorption properties of the polymer is also maximized. The interaction between diffusive selective polymers and zeolite nanoparticles is studied to improve the hybrid separation performance. Polymer materials with multifunctional chemical groups and porous fillers with hierarchical structures should be actively explored. There are relatively new polymer matrix materials, such as poly(ether-block-amide) [145] and polymers of intrinsic microporosity(PIMs) [146], which have been rarely investigated. There are numerous promising opportunities for MOFs and COFs [147] because their superiority over the commonly-used zeolites can meet the current challenges. Given the framework of MOFs, and organic ligands linking metal nodes or clusters, MOFs have larger surface areas and tunable pore sizes compared with zeolites and will be more applicable in future research. At present, various MOFs materials have been applied in separation, including ZIF-8 [148,149], Cu-BTC, Cu-BDC [150], UiO-66 [151,152], etc. Besides, membranes with inorganic materials as the bulk continuous phase should be more vigorously developed to expand the application realm of hybrid membranes in pervaporation separations. Fabrication methods should be regulated and controlled precisely. Furthermore, the priority for fabrication method designers to consider is that hybrid membranes can incorporate more fillers. Ultrathin, defect-free and hierarchical microstructures, especially in separating bulk chemicals, are urgently needed to promote the permeation flux. For separation of molecules with similar physical properties, and often similar sizes, the prerequisite of achieving both high selectivity and permeability is a much more finely-tuned three-dimensional structure. In terms of industrial application, separation of organic mixtures composed of much similar molecules in size and physicochemical properties (e.g., isomers) and separation of multicomponent organic mixtures need further discussion. Considerable efforts should be focused on membranes with high selectivity and long-term stability for future development. In addition, designing and optimizing loading density and capital of pervaporation modules with hollow fiber and spiral-wound modules and constructing the heat network into them deserve priority. Moreover, nanoparticles will be released into the feed liquid mixture, contaminating the components to be separated, if they are inappropriately embedded into membranes, and reducing the contents of nanoparticles to weaken membrane performances. In addition, hybrid processes involving membranes such as the PV-distillation process also deserve attention. Multiple opportunities for the advancement of pervaporation may include the broadening of the present status of viable application, and the utilization of new materials and fabrication techniques in membrane production. Finally, it is likely that the analysis and optimization of the energy consumption of pervaporation technology will continue being a research gap in the field that deserves the concern of scholars [153,154].

## Figures and Tables

**Table 1:** Total vapor pressure measurements of DMF + water. Reprinted with permission from ref. [25]. Copyright 1990 Elsevier.

Liquid Phase Compositions (Water)/x_a_	Total Vapor Pressure /P(kPa)	Calculated Vapor Phase Compositions (Water)/x_a_	Liquid Phase Compositions (Water)/x_a_	Total Vapor Pressure /P(kPa)	Calculated Vapor Phase Compositions (Water)/x_a_
0.0441	1.472	0.1999	0.5406	4.130	0.8664
0.1141	1.801	0.4058	0.6011	4.514	0.8972
0.1853	2.201	0.5446	0.6283	4.707	0.9097
0.2013	2.290	0.5696	0.6349	4.753	0.9126
0.2652	2.607	0.6536	0.7394	5.543	0.9514
0.3166	2.864	0.7073	0.7640	5.736	0.9585
0.3821	3.194	0.7636	0.7893	5.922	0.9652
0.4470	3.563	0.8101	0.8286	6.239	0.9741
0.4492	3.575	0.8116	0.8952	6.713	0.9859
0.4596	3.632	0.8183	0.9583	7.125	0.9946
0.4977	3.853	0.8419			

**Table 2:** Comparison of parameters of different polymeric membranes in DMF separation.

Membrane Type	Temperature (°C)	Water in Feed (wt %)	Pervaporation Flux/J × 10^2^ (kg·m^−2^·h^−1^)	Separation Selectivity/α	PSI ^a^	Ref.
PVA	25	10	1.6	17.1	27.36	[54]
		90	20.0	11.0	220.00	
PVA-g-AAm (93%)	25	10	1.3	57.7	75.01	[54]
		90	21.5	22.1	475.15	
PAN-g-PVA (93%)	25	10	0.18	21.2	3.816	[55]
		90	9.3	23.9	222.27	
PVA/PAAc	30	2.78	1.25	275	343.75	[13]
PAM/HEMA	30	0.5	2.39	464.3	1109.677	[56]
PUU-PMMA	60	20	23.1 *	6.9	159.39	[57]
		80	10.44 *	8.9	92.916	
PTFE-g-PSSA	25	10	27.7	Infinite	Infinite	[58]
Chitosan-5/PTFE	25	10	31.7	8990	284,983	[59]
NaAlg	40	0–100	26.4–120	17.4–37.8		[60]
NaAlg-g-NVP (33%)	30–50	0–100	87.1–204.6	5.6–15.4		[61]
NaAlg/PVP (25%)	40	0–100	96–181	5.5–27		[62]
PDD-TFE	50	10	7.7	1570	12,089	[63]
PI	30–60	10	3.5–38	10–70		[64]

* Pervaporation flux of DMF; ^a^ PSI = $J_{t}\cdot \alpha$.

**Table 3:** Comparison of parameters of different MMMs in DMF separation.

Membrane Type	Temperature (°C)	Water in Feed (wt %)	Pervaporation Flux (kg·m^−2^·h^−1^)	Separation Selectivity/α	PSI ^a^	Ref.
NaA/PAN	24	80	1.84	11.5	21.16	[12]
UiO-66/PI	40	10	0.1097	34.1	3.74077	[88]

^a^ PSI = $J_{t}\cdot \alpha$.

**Table 4:** Comparison of parameters of different inorganic membranes in DMF separation.

Membrane Type	Temperature (°C)	Water in Feed (wt %)	Pervaporation Flux (kg·m^−2^·h^−1^)	Separation Selectivity/α	PSI ^a^	Ref.
NaA	60	70	1.6	330	528	[131]
A-type	82	9.1	1.51	2400	3624	[124]
T-type	80	9.4	0.2	2600	520	[124]
Amorphous silica (ECN)	80	10.2	1.53	100	153	[124]
Amorphous silica (Pervatech)	80	9.1	1.14	120	136.8	[124]
Co-silicalite-1	40	95	0.66	4.4	2.094	[2]
Fe-silicalite-1	40	95	0.84	2.9	2.436	[2]
CHA	75	10	5.7	1180	6726	[134]
CHA	75	10	2.6	2000	5200	[141]

^a^ PSI = $J_{t}\cdot \alpha$.

## Data Availability

All data generated or analyzed during this study are included in this published article.

## Nomenclature

DMF, N, N-dimethylformamide; PV, Pervaporation; *c*, concentration; *K,* partition coefficient; *D*, diffusion coefficient; PSI, pervaporation separation index; *J*, permeation flux; *Q*, permeate quantity; *A*, effective membrane area; α, separation factor; *P*, permeability; *l*, thickness of the selective layer; *f*, fugacity; *x_i_*, mole fraction; *y_i_*, activity coefficient;$\;P^{sat},$ saturated vapor pressure; MFLI, membranes flash index;$\;y_{i}^{PV}$, permeate concentration; $y_{i}^{D}\left[VLE\right]$, equilibrium distillation value; MMMs, mixed matrix membranes; PVA, poly(vinyl alcohol); AAm, acrylamide; χ_ip_, membrane-solvent interaction parameter; *δs*, solvent solubility parameters; *δp*, polymer solubility parameters; PAN, polyacrylonitrile; PAAc, poly(acrylic acid); υ_c_, crosslink density; M_c_, molar mass; DMTA, dynamic mechanical and thermal analyzer; Am, arcylamid; HEMA, 2-hydroxyethyl methacrylate; PUU, polyurethane urea; PMMA, poly (methyl methacrylate); IPN, interpenetrating network; AIBN, azobisisobutyronitrile; PTFE, poly(tetrafluoroethylene); NaSS, sodium 4-styrenesulfonate; GPTMS, γ-(glycidyloxypropyl)trimethoxysilane; NaAlg, sodium alginate; NVP, N-vinyl-2-pyrrolidone; PVP, polyvinyl pyrrolidone; PDD, per-fluoro-2,2-dimethyl-1,1,3-dioxole; TFE, tetrafluoroethylene; PMDA, pyromellitic dianhydride; BTDA, 3,3′,4,4′-benzophenoneteracarboxylic dianhydride; ODA, 4,4′-diaminodiphenyl ether; MDA, diaminodiphenylmethane; BAPP, 2,2-bis [4-(4-amlnophenoxy) phenyl] propane; BZD, benzidine; DMAc, N, N-dimethylacetamide; MD, molecular dynamic; CNTs, carbon nanotubes; MWCNTs, multiwalled CNT; MOFs, metal organic frameworks; ZIFs, zeolite imidazolate frameworks; GOs, graphene oxides; POCs, porous organic cages; POSSs, polyhedral oligomeric silsesquioxanes; UiO, University of Oslo; PI, polyimide; PAA, polyamic acid; CVD, chemical vapor deposition; ALD, atomic layer deposition; CHA, chabazite; YSZ, yttria-stabilized zirconia; PIMs, polymers of intrinsic microporosity.
